# Cultural adaptation and updating of the *Risk assessment and management of exposure of health care workers in the context of covid-19* questionnaire*

**DOI:** 10.1590/1518-8345.5449.3490

**Published:** 2021-10-29

**Authors:** Maria Helena Palucci Marziale, Fernanda Ludmilla Rossi Rocha, Alex Jones Flores Cassenote, Maria Lúcia do Carmo Cruz Robazzi, Pedro Fredemir Palha, Jaqueline Garcia de Almeida Ballestero, Fábio de Souza Terra, Vivian Aline Mininel, Heloisa Ehmke Cardoso dos Santos, Isabela Fernanda Larios Fracarolli, Gracielle Pereira Aires Garcia, Maria Alice Barbosa Fortunato, Marcelo Marques de Lima

**Affiliations:** 1 Universidade de São Paulo, Escola de Enfermagem de Ribeirão Preto, PAHO/WHO Collaborating Centre for Nursing Research Development, Departamento de Enfermagem Geral e Especializada, Ribeirão Preto, SP, Brazil.; 2 Universidade de São Paulo, Departamento de Medicina Preventiva da Faculdade de Medicina da Universidade de São Paulo, São Paulo, SP, Brasil.; 3 Universidade de São Paulo, Escola de Enfermagem de Ribeirão Preto, PAHO/WHO Collaborating Centre for Nursing Research Development, Departamento de Enfermagem Materno-Infantil e Saúde Pública, Ribeirão Preto, SP, Brazil.; 4 Universidade Federal de Alfenas, Escola de Enfermagem, Alfenas, MG, Brazil.; 5 Universidade Federal de São Carlos, Departamento de Enfermagem, São Carlos, SP, Brazil.; 6 Universidade de São Paulo, Escola de Enfermagem de Ribeirão Preto, PAHO/WHO Collaborating Centre for Nursing Research Development, Ribeirão Preto, SP, Brazil.; 7 Scholarship holder at the Conselho Nacional de Desenvolvimento Científico e Tecnológico (CNPq), Brazil.; 8 Scholarship holder at the Coordenação de Aperfeiçoamento de Pessoal de Nível Superior (CAPES), Brazil.; 9 Ministério da Saúde, Unidade Técnica de Capacidades Humanas para a Saúde, Brasília, DF, Brazil.; 10 Ministério da Saúde, Departamento de Gestão do Trabalho em Saúde, Brasília, DF, Brazil.

**Keywords:** Covid-19, Occupational Health, Occupational Risks, Health Management, Health Personnel, Risk Assessment, Covid-19, Salud Laboral, Riesgos Laborales, Gestión en Salud, Personal de Salud, Medición de Riesgo, Covid-19, Saúde do Trabalhador, Riscos Ocupacionais, Gestão em Saúde, Pessoal de Saúde, Avaliação de Risco

## Abstract

**Objective:**

to translate and culturally adapt the *Risk assessment and management of exposure of health care workers in the context of covid-19* questionnaire to the Brazilian context and to develop and evaluate a sociodemographic and occupational characterization questionnaire to compose the adapted questionnaire.

**Method:**

five stages were conducted to adapt the *Risk assessment and management of exposure of health care workers in the context of covid-19* questionnaire, namely: translation, synthesis of the translations, evaluation by a committee of judges, back translation and pre-test. As for the complementary questionnaire, it was elaborated and evaluated by a committee of judges and a pre-test was carried out.

**Results:**

the questionnaires were validated and the pre-test stage was conducted with health workers and students.

**Conclusion:**

the final version adapted to the Brazilian context was called *Questionário de avaliação de risco e gestão da exposição de trabalhadores e estudantes de saúde no contexto da covid-19* and is available for use, together with the final version of the *Sociodemographic and occupational questionnaire: Risk assessment and management of exposure of health care workers and students in the context of covid-19*. These questionnaires may assist in mitigating the risks of infection, illness and death of health workers and students due to covid-19.

## Abstract (es)

**Objetivo:**

traducir y adaptar culturalmente el cuestionario *Risk assessment and management of exposure of health care workers in the context of covid-19* al contexto brasileño y elaborar y evaluar un cuestionario de caracterización sociodemográfica y ocupacional para formular el cuestionario adaptado.

**Método:**

se llevaron a cabo cinco pasos para adaptar el cuestionario *Risk assessment and management of exposure of health care workers in the context of covid-19*: traducción, síntesis de traducciones, evaluación por un comité de jueces, retrotraducción y pretest. En cuanto al cuestionario complementario, fue elaborado y evaluado por un comité de jueces y se realizó una prueba previa.

**Resultados:**

se validaron los cuestionarios y se realizó la etapa de pretest con trabajadores y estudiantes del área de la salud.

**Conclusión:**

la versión final adaptada al contexto brasileño se denominó *Questionário de avaliação de risco e gestão da exposição de trabalhadores e estudantes de saúde no contexto da covid-19* y está disponible para su uso, junto con la versión final del *Questionário de avaliação de risco e gestão da exposição de trabalhadores e estudantes de saúde no contexto da COVID-19*. Estos cuestionarios pueden ayudar a disminuir los riesgos de infección, enfermedad y muerte de los trabajadores y los estudiantes del área de la salud por covid-19.

## Abstract (pt)

**Objetivo:**

traduzir e adaptar culturalmente o questionário *Risk assessment and management of exposure of health care workers in the context of covid-19* para o contexto brasileiro e elaborar e avaliar um questionário de caracterização sociodemográfica e ocupacional para compor o questionário adaptado.

**Método:**

cinco etapas foram realizadas para adaptação do questionário *Risk assessment and management of exposure of health care workers in the context of covid-19*: tradução, síntese das traduções, avaliação por comitê de juízes, retrotradução e pré-teste. Quanto ao questionário complementar, foi construído e avaliado por um comitê de juízes e realizado pré-teste.

**Resultados:**

os questionários foram validados e a etapa de pré-teste foi realizada com trabalhadores e estudantes da área de saúde.

**Conclusão:**

a versão final adaptada para o contexto brasileiro foi denominada Questionário de avaliação de risco e gestão da exposição de trabalhadores e estudantes de saúde no contexto da covid-19 e está disponibilizada para uso, juntamente com a versão final do questionário sociodemográfico e ocupacional: avaliação de risco e gestão da exposição de trabalhadores e estudantes da área de saúde no contexto da covid-19. Estes questionários podem auxiliar na mitigação dos riscos de infecção, adoecimento e morte de trabalhadores e estudantes da área de saúde pela covid-19.

## Introduction

Health workers, as well as undergraduates in this area, suffer the various occupational risks constantly evidenced by scientific research studies carried out in different countries^(1-6)^. In Brazil, studies on occupational risks and work accidents among health professionals have resulted in a considerable updating of the scientific production^(7-13)^. With the advance of the covid-19 pandemic, caused by the *Severe Acute Respiratory Syndrome-related Coronavirus-2*(SARS-CoV-2), there was an intensification in the exposure of health workers and students to occupational risks and an excessive increase in workload. In order to categorize the risk of health professionals after exposure to patients with covid-19 and allow for the management of cases, the World Health Organization(WHO) made available, in March2020, a questionnaire entitled *Risk assessment and management of exposure of health care workers in the context of covid-19* ^(14)^. The WHO recommends the use of this questionnaire by health institutions providing care to patients with covid-19 and that it should be answered by all health workers exposed to the new coronavirus.

Given the need for the managers of health institutions to mitigate the occupational risks related to the pandemic caused by covid-19, the cultural adaptation of the *Risk assessment and management of exposure of health care workers in the context of covid-19* questionnaire to the Brazilian context was conceived. The procedures related to the cultural adaptation of exploratory, clinimetric and psychometric questionnaires represent face and content validation stages, which must be conducted with the main objective of evaluating the meaning and relevance of the questionnaire items and their ability to assess the construct proposed with quality^(15-16)^.

Based on the advances in scientific knowledge regarding the prevention of the infection and occupational risks related to covid-19 and the management of contamination cases in health workers, a need to streamline the questionnaire made available by the WHO is also identified. Thus, the proposal was to prepare a complementary questionnaire which, in addition to identifying and characterizing the health workers and students, proposes to characterize lifestyle, the working conditions, the adoption of individual and collective safety and protection measures in the workplaces and the existence of institutional protocols adopted by the services, aimed at protecting the workers’ and students’ health and other risk factors present in the work environments. It is also noteworthy that, in documents from the Pan American Health Organization(PAHO) and from the Brazilian Ministry of Health(*Ministério da Saúde*,MS), related to the coping strategies for covid-19, senior health students are included in the category of health professionals.

## Objective

To translate and culturally adapt the *Risk assessment and management of exposure of health care workers in the context of covid-19* questionnaire to the Brazilian context and to develop and evaluate a sociodemographic and occupational characterization questionnaire to compose the adapted questionnaire.

## Method

### Study design

This is a methodological study, carried out to translate and culturally adapt the *Risk assessment and management of exposure of health care workers in the context of covid-19* ^(14)^ questionnaire to the Brazilian context and to develop and evaluate a sociodemographic and occupational characterization form to compose the adapted questionnaire.

### Questionnaire

The *Risk assessment and management of exposure of health care workers in the context of covid-19* ^(14)^questionnaire was developed by the WHO, originally in English, and is freely accessible, under license CCBY-NC-SA3.0 IGO, which reserves the right to copy and redistribute the material in any medium or format and adapt it, remix it, transform it and even create other materials from it. It consists of a section entitled “*Covid-19 virus exposure risk assessment form for HCWs*”, which aims at categorizing the risk to which the workers caring for patients with covid-19 are exposed and is divided into seven topics related to the worker’s characterization data, contact history and interactions with patients with covid-19, activities performed, adherence to individual and collective protection procedures in the provision of health care, and accidents with biological material. At the end of these topics, the item called “*Risk categorization of health workers exposed to covid-19 virus*” shows the risk classification of the health workers exposed to the new coronavirus. Subsequently, there is another section entitled “*Management of health workers exposed to covid-19 virus applies only to exposure in health care settings*” with the objective of instructing the workers about the main actions to be taken regarding exposure to patients with covid-19, based on the prior identification of risk and case management actions to the health managers^(14)^.

### Procedures

The process of cultural adaptation of this questionnaire to the Brazilian context followed the recommendations of the WHO Translation Protocol^(14,17-22)^: 1)translation; 2) synthesis of the translations; 3) evaluation by the Committee of Judges; 4) back translation; 5) pre-test and elaboration of the final version.

In the translation stage, two independent translations into the target language are recommended, made by two different and qualified translators, preferably native speakers. This action aims at the perception of the discrepancies that may reflect a text with more than one meaning in the original language^(22)^. The *Risk assessment and management of exposure of health care workers in the context of covid-19* questionnaire was translated according to the aforementioned recommendations, resulting in the versions translated into the Portuguese language spoken and written in Brazil in two versions: Portuguese Version1(PV1) and Portuguese Version2(PV2). For the synthesis of the translations, a third translator analyzed both versions, acting as a mediator of the differences and producing the Consensus Version in Portuguese1(CVP1).

With these three versions in hand, the evaluation by the committee of judges was carried out to analyze the idiomatic (analysis of the meaning of idioms), semantic (interpretation of the meaning of words, concepts and expressions), cultural (adaptation of words, concepts and expressions to the cultural reality in which the questionnaire will be used) and conceptual(correspondence of the original concepts to those culturally accepted in the context to be studied) equivalences of the questionnaire items, verifying the need for adaptations^(15-16,22)^. The committee of judges was composed of nine health professionals, bilingual, with experience in the subject matter under study and/or working in services aimed at assisting patients with covid-19 during the pandemic, selected by inviting the groups of health professionals from the social networks. It consisted of a physician specialized in epidemiology and viral infection; two physiotherapists, PhDs in Sciences and with experience in Workers’ Health and in the care of critically-ill patients; two nurses, MScs in Science, working in treatment units for patients with covid-19; a nurse specialized in Workers’ Health, PhD in Sciences; and a pharmacist, also PhD in Sciences. Additionally, two senior medical students joined the team. The committee meeting was held remotely and included an assessment of the pertinence, adequacy and clarity of the items and answer options of the three translated versions of the questionnaire, in addition to the analysis of the cultural equivalence of the texts. The judges’ answers and suggestions were analyzed and accepted, and the result was the Consensus Version in Portuguese2(CVP2), called *Avaliação de risco e gestão da exposição de profissionais de saúde no contexto da covid-19*.

The back translation was performed to ensure that the adapted version of the questionnaire would faithfully reflect the original version. Thus, CVP2 was back-translated by two bilingual translators into the original language of the questionnaire(English), blindly, that is, without prior knowledge of the concepts already explored^(22)^, obtaining English Version1(EV1) and English Version2(EV2). A third translator analyzed the differences found and the Consensus Version in English(CVE) was generated and forwarded to the WHO for consent and final approval.

At the same time, a sociodemographic and occupational questionnaire was elaborated to complement data collection on the risk and infection factors of health professionals and undergraduates in the care of patients with covid-19. Its construction was based on current scientific evidence on the occupational risks, infection prevention and management of the cases of SARS-CoV-2 contamination in health workers^(23-26)^.

The questionnaire was submitted to the evaluation of a committee of judges, composed of six bilingual researchers: three nurses, one of whom was a PhD and a specialist in Workers’ Health and two being MScs, specialists in Psychiatric and Public Health Nursing, respectively, working on the front line of the care provided to patients with covid-19; two were physiotherapists, PhDs, specialists in Workers’ Health; and an infectious disease physician, PhD and supervising residents acting on the front line of care. The judges analyzed the appearance, clarity/understanding, content, objectivity, possible difficulties of the respondents and the time they needed to complete the questionnaire, in two stages, so that all the suggestions could be analyzed and adapted by the researchers. These judges were selected by consulting the Directory of Researchers of the National Council for Scientific and Technological Development(CNPq), due to their experiences in research. The result of this process was the elaboration of the final consensus version of the questionnaire. It is noteworthy that the informed consent from all the participants of the two groups of the expert committee was obtained, by means of the Free and Informed Consent Form(FICF), created specifically for these two stages of the study.

### Data analysis

To analyze the adequacy of both tools(the *Risk assessment and management of exposure of health care workers in the context of covid-19* questionnaire and the sociodemographic and occupational questionnaire), the Content Validity Index(CVI) was used, which measures the proportion or percentage of the judges’ answers that are in agreement with certain aspects of the questionnaire items^(27)^. To assess relevance and representativeness, the answers can include the following: 1=not relevant or not representative; 2=the item needs major review to be representative; 3=the item needs minor review to be representative; and 4=relevant or representative item. The index score is calculated by adding up the items that received “3” or “4” answers by the evaluators. The items that scored “1” or “2” must be reviewed or deleted. To be considered valid, the CVI of the questionnaire must be greater than0.78^(27-28)^. Thus, items rated1 or2 were reviewed or deleted and received no score; and those rated3 or4 were considered as valid answers, reviewed(if necessary) and received a score of1. The valid answers were added up and divided by the total number of nine judges, characterizing the CVI of the items(Formula1). The CVI of the questionnaire was calculated from the product of the sum of the items’ CVI and the total number of items in the questionnaire(Formula2)^(29)^:

Formula 1: $$\text{ IVC item}=\frac{\sum \text{ valid answers}}{\sum \text{ judges}}$$

Formula 2: $$\text{ IVC instrument}=\frac{\sum \text{ IVC items}}{\sum \text{ number of items}}$$

### Setting and population

The pre-test represents the final stage of the cultural adaptation methodological process, in which the questionnaire must be evaluated by a sample of the target population, consisting of 30 to 40 individuals^(18,22^. Thus, the consensus versions in Portuguese of both questionnaires were evaluated by health workers and undergraduates working on the front line of care against covid-19, from different Brazilian regions. The following were considered as inclusion criteria: workers and students belonging to the professional categories of physicians, nurses, nursing technicians and assistants, physiotherapists and dentists, and who worked in the front line of care for patients with covid-19. Thus, were invited to participate in this stage 382 workers and undergraduates, linked to research groups in the areas of Workers’ Health, Public Health and Intensive Care, as well as post-undergraduates and graduates of the universities participating in the study. Of them, 34 fully answered the questionnaire and comprised the sample for this stage of the study. The workers and students who did not answer the questionnaires were excluded, as well as those who answered them partially.

Recruitment to participate in the pre-test was done by sending an invitation via email and/or messages(*WhatsApp*), with a maximum of three attempts being made to obtain the answers, and with an interval of 10 days between contacts. Those who agreed to participate in the research were sent an individual and non-transferable link with agreement expressed in a Free and Informed Consent Form(FICF) referring to the two data collection tools, along with a form with five open questions about the understanding and relevance of the items and about the adequacy of the format and answer options for the respondents. The mean time to fill out both tools was 25 minutes, both for health professionals and students.

The adjustments resulting from this stage were implemented by the researchers, which resulted in the final versions of the questionnaires: 1)Final Version in Portuguese(FVP) of the *Risk assessment and management of exposure of health care workers in the context of COVID-19* questionnaire, translated and adapted to the Brazilian context for health workers and students, called *Questionário de avaliação de risco e gestão da exposição de trabalhadores e estudantes de saúde no contexto da covid-19*; and 2)final version of the *Sociodemographic and occupational questionnaire: Risk assessment and management of exposure of health care workers and students in the context of covid-19*.

### Ethical aspects

The study was approved by the Research Ethics Committee of the Ribeirão Preto Nursing School at the University of São Paulo(*Comitê de* Ética *em Pesquisa da Escola de Enfermagem de Ribeirão Preto da Universidade de São Paulo*,CEPEERP/USP) (No.0208/2020). The recommendations set forth in Resolution466/2012 referring to the ethical standards for research involving human beings^(30)^ and in the General Data Protection Law-GDPL^(31)^ were followed.

## Results

During the process of translating and culturally adapting the *Risk assessment and management of exposure of health care workers in the context of covid-19* questionnaire to the Brazilian context, changes were made to the content of the questions in item1 so that the questionnaire could be self-administered, facilitating data collection by researchers or health service managers, which resulted in the exclusion of items related to the interviewer’s data. In order to improve the allocation of items related to occupational exposure to patients infected with SARS-CoV-2 and to facilitate the respondents’ understanding, questions3 and4 of the original questionnaire were modified. The statements in questions5 and6 did not undergo significant changes. However, updates were introduced regarding the types of Personal Protective Equipment(PPE) to be used by the workers during care for patients with covid-19, aiming to adapt them to the current pandemic context. Originally, the questionnaire presents the following PPE: *single-use gloves, N95 mask(or equivalent respirator), face shield or goggles/protective glasses, disposable gown, waterproof apron* and, in the final version adapted for the Brazilian context, the following were detailed: *disposable gloves, N95 mask(or equivalent respirator), shield/face shield or goggles, disposable apron, waterproof apron and disposable cap*, in accordance with internationally accepted recommendations and up-to-date scientific evidence.

Updates related to the guidelines for the health workers regarding exposure to patients with covid-19 and the management of cases by health managers were implemented, since the original questionnaire was dated March2020, the beginning of the pandemic, when the scientific evidence about the collective and individual prevention measures for the workers’ health were scarce, such as exposure, main forms of contamination and management of confirmed cases, among others. Therefore, questions about testing for covid-19 were included in the complementary questionnaire; as well as referring to quarantine; self-monitoring of body temperature and respiratory symptoms; communication to the head of the health service about any symptoms suggestive of covid-19; adoption of the contact and droplet precautions during care for all patients with acute respiratory diseases and of the standard precautions for all patients; adoption of the environmental precautions for aerosol-generating procedures in all patients with a suspected or confirmed covid-19 diagnosis; rational and correct use of PPE; hand hygiene before touching a patient, before any procedure, after exposure to bodily fluids, after touching a patient and/or areas close to the patient; practice of respiratory etiquette, psychosocial support to the health workers during the pandemic, quarantine or during the period of the covid-19 disease; respect for the legislation in force regarding the remuneration of workers during the pandemic; review and adequacy of protocols for the procedures, organizational, clinical and treatment flows, and training on infection prevention and control for all health workers and undergraduates.

Regarding the Content Validity Index of the adapted questionnaire, the items related to the information about the interviewer(1 A and 1 C) presented CVI values of0.33 and0.22, respectively, being considered of little relevance or irrelevant and, therefore, they were deleted. The other items were considered very or highly relevant and presented CVI values between 0.89 and 1.00, being considered as valid answers. The questionnaire obtained a total CVI=0.95.

The *Sociodemographic and occupational questionnaire: Risk assessment and management of exposure of health care workers and students in the context of covid-19* was constructed with 48 questions related to the participants’ characterization, divided into eight sections: I.Professional Identification, II. Clinical Characterization, III. Family/Residence Characterization, IV. Lifestyle, V.Work Characterization, VI. Safety at work, VII. Institutional protocol in case of an infected health professional and/or student, and VIII. Other Risk Factors. During its elaboration and evaluation, it was observed that, after the first evaluation stage, the questionnaire presented a CVI=0.95, considered adequate. However, there was a need to exclude the following questions: “*Você está aposentado?*” (Are you retired?) (Item’s CVI=0.71), “*Aposentado há quanto tempo?*” (How long?) (Item’s CVI=0.71), “*Como você se declara em relação a sua cor ou raça/etnia?*” (How you declare yourself in relation to your race/ethnicity?) (Item’s CVI=0.71), “*Pratica alguma crença religiosa?*” (Do you practice any religious beliefs?) (Item’s CVI=0.43) and “*Qual religião?*”(Which one?) (Item’s CVI=0.43). After adaptation, according to the evaluators’ suggestions, a new evaluation round was carried out.

In this second stage of analysis, after removing the aforementioned questions, items related to pre-existing diseases, to the need for isolation, and to the frequency and practice of physical exercises were added. After a new analysis by the evaluators, the CVI of the items reached values above0.83 and the final CVI of the complementary questionnaire obtained a score of0.97. Thus, the final consensus version of the *Sociodemographic and occupational questionnaire: Risk assessment and management of exposure of health care workers and students in the context of covid-19* was prepared, which was attached to CVP2 of *Questionário de avaliação de risco e gestão da exposição de trabalhadores e estudantes de saúde no contexto covid-19*. The versions were applied in the pre-test to a sample of 34 workers and students attending the last year of undergraduate health courses, consisting of 11 men and 23 women, aged between 22 and 62 years old(mean of 36.4±11.9years old; median of 33 years old), from all Brazilian regions, with the following distribution: two from the North(Acre); 11 from the Northeast(eight from Ceará; two from Pernambuco and one from Rio Grande do Norte); seven from the Midwest(Goiás); 11 from the Southeast(five from Minas Gerais, one from Rio de Janeiro and five from São Paulo) and three from the South(two from Paraná and one from Rio Grande do Sul). Regarding the professional categories, they were as follows: 16 nurses, eight physiotherapists, six physicians, two Nursing undergraduates, a nursing technician and a dentist.

The evaluations by the participants allowed the following adjustments to be made to the sociodemographic and occupational questionnaire and to the operationalization of data collection from the participants: inclusion of the undergraduate student option in the “*Categoria Profissional*” (“Professional Category”) question, in the item called “*Informações do Profissional de Saúde*” (“Health Professional Information”); establishment of questionnaire completion if the participants answered negatively questionsA, B, C andD in the item called “*Informações sobre interações do profissional para a prestação de cuidados de saúde aos pacientes com covid-19*” (“Information about interactions of the professional for the provision of health care to patients with covid-19”); option to maintain the absence of the “*obesidade*” (“obesity”) answer among the pre-existing diseases, due to the possibility of using the Body Mass Index(BMI); adequacy of the open fields according to the specifications of the questions, for example, only numbers in the questions referring to weight and height, among others; changes in the answer options for the “*Formas de isolamento*” (“Forms of isolation”) question, including the alternatives “*Mudou de domicílio ou as outra(s) pessoa(s) mudaram de domicílio*” (“Changed address or the other person/people changed address”); “*Isolado em um quarto/cômodo, separado das outras pessoas*” (“Secluded in a bedroom/room, separated from the other people”) and “*Outra. Especificar*” (“Other. Specify”); adequacy of the questions related to the routine testing for covid-19 in the health services where the health professionals and students work; verifying the best time for recruiting the participants and forwarding the access link to fill in the questionnaire; and creation of a non-individualized link, allowing access by multiple participants and individual reporting of the data completed. We emphasize the development and implementation of two questions related to the strategies for the vaccination of health workers, such as receiving the vaccine, type of vaccine, number and interval of doses received and occurrence of adverse events after vaccination, complementing the introduction of updated scientific evidence about the safety and protection measures for health workers and students in the context of the covid-19 pandemic.

## Discussion

The face and content validation process of the questionnaires was carried out based on robust and internationally accepted methodological frameworks^(17,19,22,24)^, enabling the rigorous elaboration and adaptation of important tools for the mitigation and management of occupational risks in the Brazilian health services in the context of covid-19 pandemic.

The methodological procedures performed for the *Risk assessment and management of exposure of health care workers in the context of covid-19* questionnaire represented not only its translation and cultural adaptation to the Brazilian context, but also the updating of fundamental aspects related to the prevention of risks and the management of the workers’ health problems caused by covid-19^(27-29)^, released after the construction by the WHO of the aforementioned questionnaire. In this sense, recommendations were introduced on individual and collective actions aimed at preventing the contamination of health professionals during the care activities for patients with covid-19, based on measures recommended by the Centers for Disease Control and Prevention^(28)^ and by the WHO^(27,29)^, among which the following stand out: need for hand hygiene with water and liquid soap and/or 70% alcohol preparation and correct use of PPE[protective glasses or face shield, surgical mask, waterproof apron and procedure gloves; cap and N95 or Filtering Face Piece type2(FFP2) mask during aerosol-generating procedures, such as tracheal intubation or aspiration, manual ventilation before intubation, and use of mechanical ventilation, cardiopulmonary resuscitation, and nasotracheal specimen collections]. A study presents evidence that supports these recommendations for the individual protection of workers^(32)^.

In relation to the collective actions for the protection, prevention and control of occupational contamination in the pandemic context, the measures recognized by the WHO were followed^(29)^ and discussed in a study on minimizing the impact of the epidemic on the health systems^(33)^. These measures were evidenced in a narrative literature review study that reinforces the importance of social distancing, the quarantine of suspected or confirmed cases and restrictions to contain the spread of the virus, such as adaptations for the use of public transportation and the installation of sanitary barriers in the cities^(34)^.

Complementing the recommendations for the Brazilian context to fight against the covid-19 pandemic, it is considered fundamental to effectively record the confirmed cases in the information systems; to review the care flows and the adequacy of the working conditions in the health services; to adopt care actions for the workers’ health, with special attention to reducing workloads and stress in the work environments; and to establish covid-19 as a work-related disease^(35)^.

Regarding the use of the *Risk assessment and management of exposure of health care workers in the context of covid-19* questionnaire, it was verified that it was used in a research study conducted in Saudi Arabia, with the objective of evaluating the post-contact risk of nurses who provided assistance to patients diagnosed with covid-19^(36)^. The questionnaire was applied to a sample of 80 nurses working in hospitals located in the North of the country, and it was identified that 8.8% of the workers had a high risk of failure in the removal and exchange of the PPE; that 6.3% had a high risk of not performing hand hygiene before and after touching patients with covid-19; and that 5% did not follow the recommended guidelines for hand hygiene after touching the patients’ surroundings. In addition to that, 3.8% of the participants suffered accidents related to biological material, such as biological fluid splashes(in the eyes) and were classified as at high risk for infection by the covid-19 virus.

In Bangladesh, the WHO questionnaire was used to determine the role of the individual protection measures in preventing the spread of covid-19 among physicians with positive and negative diagnoses of the disease and among those who worked in different health units^(37)^. Among the main findings, it was evidenced that the frequent decontamination of the environment and the use of face shields/protective glasses and N95 masks during the performance of aerosol-generating procedures in patient care represented protective actions against covid-19; and that the physicians who reused garments presented twice the chances of being covid-19 positive than those who did not. In Egypt, a study using the WHO questionnaire had as its objective to assess the risk and management of the health workers’ exposure in the context of covid-19^(38)^ in a sample of 230 professionals. The results revealed high risk of contamination for three specific groups, namely: health workers who did not use PPE during the care of infected patients(20%); health workers who used it, but not in all procedures or contacts with the contaminated patient environment(20%to35%); and health workers who suffered accidents with exposure to biological materials during interactions with patients with covid-19(34%).

Although it was found that the use of the *Risk assessment and management of exposure of health care workers in the context of covid-19* questionnaire allows for detailed information collection on occupational risks and the management of the exposure of health workers and students to SARS-CoV-2, the performance of procedures for its cultural adaptation to the different countries mentioned above was not identified. No other studies that used this questionnaire for data collection were identified, nor was its use identified in nationwide surveys.

Regarding the *Sociodemographic and occupational questionnaire: Risk assessment and management of exposure of health care workers and students in the context of covid-19*, the face and content validation process proved to be fundamental for the adequacy of the questions and answer options related to the individual and occupational characterization of the participants and to the working conditions faced by the workers and students, in the pandemic context.

In this sense, it is considered that this study enabled the availability of an important tool for assessing occupational risks and managing the exposure of health workers and students to SARS-CoV-2 in Brazil. Assessments of both questionnaires will be obtained from a future research study, which is being carried out by the group of researchers in a sample of health workers and university students who participate in the “*O Brasil Conta Comigo*” (“Brazil Counts on Me”) program of the Brazilian Ministry of Health, in the states of Amazonas, Roraima and Amapá by means of the AGIR-COV-2020 Project (https://sites.usp.br/agir/)

In addition, it is considered that the publication of the questionnaires now available and properly validated in terms of face and content, disclosed through this article for immediate use by health service managers, is necessary in view of the alarming number of infected Brazilians, among which are thousands of health workers and students. It is noteworthy that Brazil already records more than 13.4 million people infected with SARS-CoV-2, and that at least 1,200 physicians and nursing professionals have already died as a result of covid-19 since March last year, according to the class entities^(39)^. This situation has overloaded the health systems of the countries most affected by the pandemic, generating exhaustion in the professionals working on the front lines and physical and mental illness in the workforce^(40)^.

Thus, the implementation of recommendations related to individual and collective measures to protect the workers’ health and the management of the occupational exposure to SARS-CoV-2 are of paramount importance to update and complement the WHO questionnaire, increasing its effectiveness for the collection of diverse information about the health and working conditions of health professionals and students acting on the front line of health care for patients with covid-19, at different care levels.

## Conclusion

This study originated the final version of the *Risk assessment and management of exposure of health care workers in the context of covid-19* questionnaire, translated and adapted to the Brazilian context for health workers and students, called *Questionário de avaliação de risco e gestão da exposição de trabalhadores e estudantes de saúde no contexto da covid-19* and, in conjunction with the application of the *Sociodemographic and occupational questionnaire: Risk assessment and management of exposure of health care workers and students in the context of covid-19*. The methodological rigor adopted in all stages of the study provides reliability to the face and content validation process of the questionnaires and allows its use for risk assessment and management of the exposure of health professionals and students in the context of covid-19, so that, based on the identification of occupational risks for infection by SARS-CoV-2, health service managers can plan preventive actions against the illness of these professionals and students.
